# Influence of Solvent Polarity and DNA-Binding on Spectral Properties of Quaternary Benzo[*c*]phenanthridine Alkaloids

**DOI:** 10.1371/journal.pone.0129925

**Published:** 2015-06-19

**Authors:** Michal Rájecký, Kristýna Šebrlová, Filip Mravec, Petr Táborský

**Affiliations:** 1 Central European Institute of Technology (CEITEC), Masaryk University, Brno, Czech Republic; 2 Department of Biochemistry, Faculty of Medicine, Masaryk University, Brno, Czech Republic; 3 Materials Research Centre, Faculty of Chemistry, Brno University of Technology, Brno, Czech Republic; 4 Department of Chemistry, Faculty of Science, Masaryk University, Brno, Czech Republic; University of Quebec at Trois-Rivieres, CANADA

## Abstract

Quaternary benzo[*c*]phenanthridine alkaloids are secondary metabolites of the plant families *Papaveraceae*, *Rutaceae*, and *Ranunculaceae* with anti-inflammatory, antifungal, antimicrobial and anticancer activities. Their spectral changes induced by the environment could be used to understand their interaction with biomolecules as well as for analytical purposes. Spectral shifts, quantum yield and changes in lifetime are presented for the free form of alkaloids in solvents of different polarity and for alkaloids bound to DNA. Quantum yields range from 0.098 to 0.345 for the alkanolamine form and are below 0.033 for the iminium form. Rise of fluorescence lifetimes (from 2–5 ns to 3–10 ns) and fluorescence intensity are observed after binding of the iminium form to the DNA for most studied alkaloids. The alkanolamine form does not bind to DNA. Acid-base equilibrium constant of macarpine is determined to be 8.2–8.3. Macarpine is found to have the highest increase of fluorescence upon DNA binding, even under unfavourable pH conditions. This is probably a result of its unique methoxy substitution at C_12_ a characteristic not shared with other studied alkaloids. Association constant for macarpine-DNA interaction is 700000 M^-1^.

## Introduction

Quaternary benzo[c]phenanthridine alkaloids (QBAs) are secondary metabolites of some species of the plant families *Papaveraceae*, *Rutaceae*, and *Ranunculaceae*. The most abundant representatives are commercially available sanguinarine and chelerythrine. Isolations of other QBAs, namely sanguilutine, sanguirubine, chelilutine, chelirubine, and macarpine, were reported by Slavik *et al*. [1–3]. The source of these rare alkaloids has been plant material [4,5] even though the synthetic approach was published for macarpine [6].

**Fig 1:**
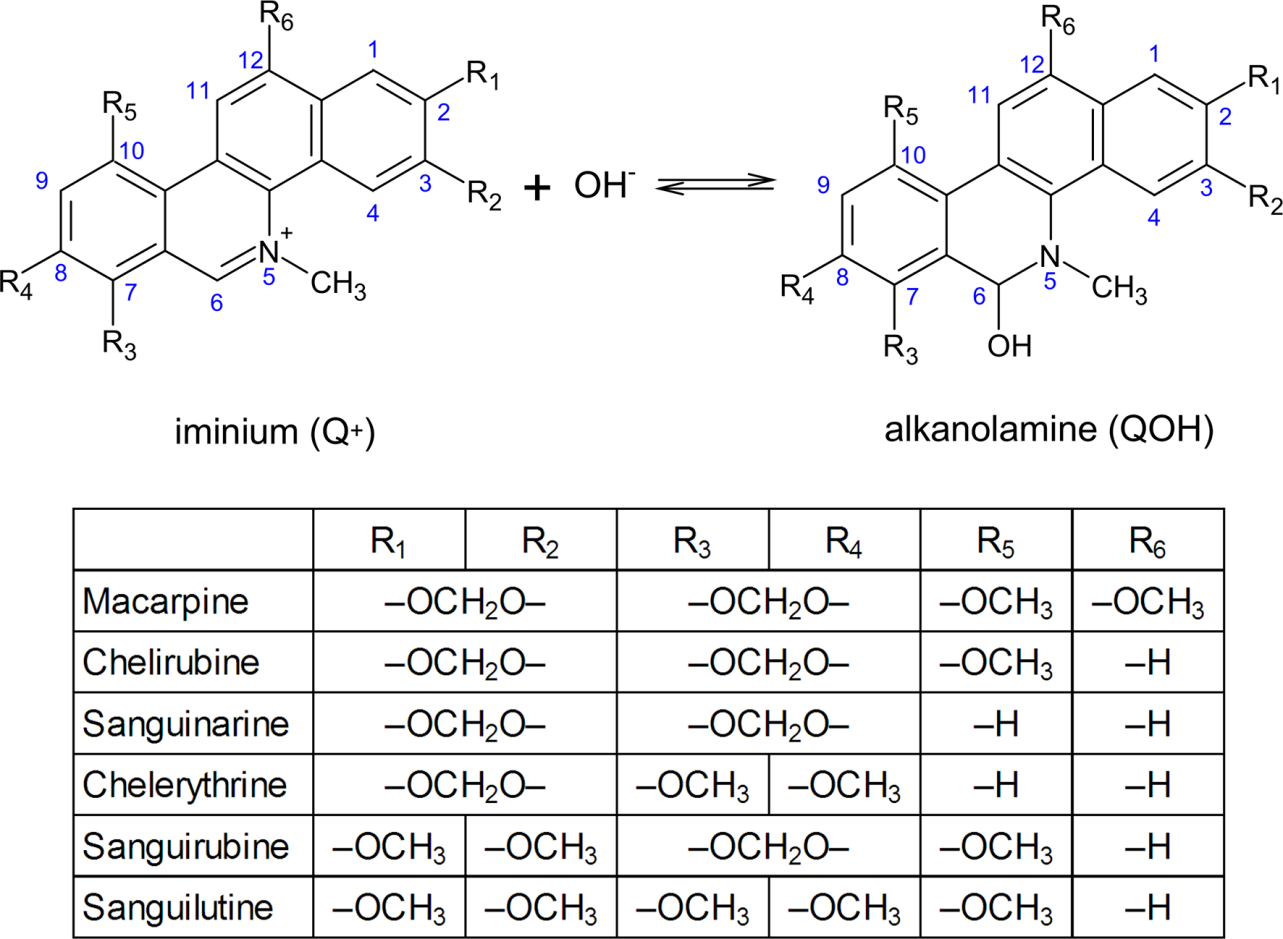
Structure, numbering and acid-base equilibrium of studied QBAs.

QBAs are composed of a *N*-methylbenzo[*c*]phenanthridinium core with several methoxy or methylenedioxy substituents (Fig 1). Due to the reactive iminium bond, QBAs are susceptible to nucleophilic addition on carbon C_6_ [7]. Therefore, equilibrium between the iminium and alkanolamine forms is established in aqueous solution (Fig 1).

The biological effects of QBAs that have been studied were mainly for sanguinarine and chelerythrine. Anti-inflammatory, antifungal, antimicrobial activities and possible anticancer effects of QBAs have been reported (reviewed in [8,9]). At the molecular level, interactions with various forms of DNA, proteins and enzymes have been reported for sanguinarine and chelerythrine [10–16]. The properties of other QBAs are less known and have been studied mainly at cellular level [17,18]. Their ability to quickly enter into cells has been used for cell staining [19].

While the fluorescence of sanguinarine and chelerythrine is well described [20–23], knowledge of the fluorescence properties of other QBAs is limited. Fluorescence spectra in aqueous solution and their change upon binding to DNA has previously been reported [24] and their potential use as probes in fluorescence microscopy and flow cytometry was proposed [19]. A detailed description of the fluorescence of iminium and alkanolamine forms of QBAs has not yet been performed. Spectral changes are an important indicator of the microenvironment around a fluorescent probe. Sensitivity to solvent polarity could help to explain the mode of binding to biomacromolecules especially when no structure of such complexes is available. Additional information could be provided by fluorescence lifetimes. Therefore, fluorescence properties of both iminium and alkanolamine forms of QBAs (except chelilutine) in solutions of different polarity and upon binding to DNA are presented.

## Materials and Methods

### Quaternary benzo[*c*]phenanthridine alkaloids and chemicals

All studied alkaloids were isolated from plant material as published earlier [25] and were obtained as chloride salts of at least 93% purity. Stock solutions were prepared by dilution in MilliQ water (Millipore, USA) and were stored at room temperature in the dark. Trizma base, ethylenediaminetetraacetic acid disodium salt (EDTA), quinine, colloidal silica (Ludox), calf thymus DNA (ctDNA), and salmon testes DNA were purchased from Sigma-Aldrich, USA. All other chemicals were obtained from Lach-ner, Czech Republic. The ctDNA was dissolved in 10mM Tris–1mM EDTA buffer, pH 7; salmon testes DNA was dissolved in MilliQ water. The concentration of DNA was determined spectrophotometrically using the relationship that 1 absorbance unit at 260 nm corresponds to 50 μg ml^-1^ (0.075 mM in base-pairs (bp)) of double-stranded DNA.

### Absorbance and fluorescence measurements

Absorbance was measured on a Shimadzu UV-1601 spectrophotometer (Shimadzu, Japan). Steady-state fluorescence was measured on an Aminco Bowman Series-2 spectrofluorometer (SLM Aminco, USA). All measurements were performed at 25°C. If not stated otherwise, fluorescence spectra were corrected for lamp instabilities and non-ideal instrument detection. Validity of corrected spectra produced by correction factors supplied by the manufacturer of the spectrofluorometer was assessed by comparison with corrected spectra of fluorescent probes published by Lakowicz [26]. When necessary, spectra were converted to a wavenumber scale using Eq (1), where *I* denotes intensity at a particular wavenumber (*ν᷉*) or wavelength (*λ*), to take non-constant bandpass in wavenumber scale into account. Spectra in wavenumber scale were smoothed by a Savitzki-Golay filter using 2^nd^ order polynomial over 11 points.

$$I(\tilde{\nu })=I(\lambda )\lambda^{2}$$ (1)

### Acid-base behaviour of macarpine

Acid-base equilibrium of a QBA (Fig 1) is defined by the equilibrium constant K_ROH_ (Eq (2), also denoted as K_R+_) that is connected to a common acid-base equilibrium constant K (Eq (3), Fig 1) by a water ion product K_ROH_ = K∙K_W_ [27,28]. Negative logarithm of K_ROH_ is analogous to pK_a_ of common acids and will be marked pK_ROH_ in this article.

$$K_{ROH}=\frac{\left[H^{+}][QOH\right]}{\left[Q^{+}\right]}$$ (2)

$$K=\frac{\left[QOH\right]}{\left[Q^{+}][OH^{-}\right]}$$ (3)

Samples were prepared by diluting macarpine stock solution to 3 μM by Britton-Robinson universal buffer with a pH between 3 and 12 and ionic strength 0.15 M [29]. Absorption and fluorescence spectra were recorded as described above. Measurements were performed 3 times and mean values of absorbance at 495 nm and fluorescence at 450 nm were fitted to Eq (4) [30]

$$A=\frac{A_{1}+A_{2}\cdot 10^{pH-pK_{ROH}}}{1+10^{pH-pK_{ROH}}}$$ (4)

where *A* is the signal (absorbance or fluorescence) at a particular pH, *A*_*1*_ is the signal of iminium form, *A*_*2*_ is signal of the alkanolamine form and *pK*_*ROH*_ is the constant defined above. QtiPlot 0.9.8.9 was used for weighted fitting with variance used as a weight. Standard errors of best-fit parameters (SE) are reported in Table 1.

**Table 1:** Spectroscopic properties of alkanolamine (QOH) and iminium (Q^+^) forms of QBAs.

				Fluorescence lifetime (ns)^e^					
				pH 9.45 (QOH)		pH 3.95 (Q^+^)		S_1_→S_0_ rate const. of QOH (10^7 ^s^-1^)	
Alkaloid	pK_ROH_ ^a^	QY QOH^d^	QY Q^+ ^ ^d^	no DNA	ctDNA	no DNA	ctDNA	k_r_^S^	k_nr_^S^
sanguinarine	8.05	0.210	0.033	3.2^f^	3.2	2.4^i^	2.4 (10.1)	6.56	24.69
chelerythrine	9.0	0.156	0.004	3.2	3.2	—	3.1 (10.3)	4.88	26.38
sanguilutine	8.8	0.208	—	3.5	3.5	—	3.8	5.94	22.63
sanguirubine	7.9	0.233	—	3.9	3.9	—	2.9	5.97	19.67
chelirubine	7.7	0.345	—	4.5	4.5	—	3.1	7.67	14.56
macarpine	8.24^b^ (8.30^c^)	0.098	—	3.9	3.3^g^ (4.6^h^)	—	3.1 (5.1)	2.51	23.13

Where two results were obtained (pK_ROH_ by two methods, two lifetimes), second value is indicated in brackets.

a) all QBAs except macarpine ref. [30].

b) this work, spectrophotometry, n = 3, SE = 0.02

c) this work, spectrofluorometry, n = 3, SE = 0.06

d) n = 4, estimated RSD ca. 15%

e) n = 3, SE ≤ 0.1 ns

f) 3.3 ns (ref. [22])

g) 3.5 ns (610 nm)

h) 5.8 ns (610 nm)

i) 2.4 ns (ref. [22])

### Macarpin binding to DNA

Measurements were performed in 0.05 M citrate buffer, pH 6.15, containing 0.122 M Na^+^. A set of samples with a constant macarpin concentration 1 × 10^-5^ M and varying concentrations of salmon testes DNA were prepared. Absorption and fluorescence spectra were measured as stated above. Inner-filter effect was corrected using Eq (5) [31], where *F*_*corr*_ is fluorescence corrected for inner-filter effect, *F*_*meas*_ is measured fluorescence, *A*_*ex*_ is absorbance at excitation wavelength, *A*_*em*_ is absorbance at emission wavelength, and *d*_*ex*_ and *d*_*em*_ are dimensions of light path in excitation and emission direction, respectively.

$$F_{corr}=F_{meas}\cdot 10^{\left(A_{ex}\cdot d_{ex}/2+A_{em}\cdot d_{em}/2\right)}$$ (5)

DynaFit program [32] was used to analyse binding of macarpin to double-stranded DNA by observing corrected fluorescence at 625 nm as a function of the total DNA concentration and conditional association constant for 1:1 binding was obtained. Measurements were performed 3 times and standard deviations (SD) for small samples were calculated according to Dean and Dixon [33].

### Fluorescence lifetimes

QBAs in 3 × 10^-6^M concentration were prepared in 0.1M borate buffer, pH 9.45 (alkanolamine form) or in 0.1M acetate buffer, pH 3.95 (iminium form). For measurement of lifetimes of QBA-DNA complexes, ctDNA was added (DNA base pair:drug ratio 1.6:1). Measurements were performed on a Fluorocube (Horiba Jobin Yvon, France) with a 329 nm excitation LED diode (pulse width 1.2 ns) and emission monochromator set to 440 nm (alkanolamine form) or 610 nm (iminium form, 570 nm for chelerythrine). Instrument response function was obtained by measuring a solution of colloidal silica. When necessary, intensity was lowered by neutral filters. For each QBA three measurements were performed and lifetimes were globally fitted using DecayFit 1.3. Quality of a fit was considered by inspecting residuals and reduced χ^2^ value around 1. SE are reported in Table 1.

### Quantum yields

Quinine sulfate dissolved in 0.1M H_2_SO_4_ and anthracene dissolved in ethanol for spectroscopy were used as standards. Alkanolamine forms of QBAs were measured in 0.01M borate buffer, pH 9.45. Iminium forms of sanguinarine and chelerythrine were measured in 0.01M acetate buffer, pH 3.95. Five to 8 solutions with absorbances at 322 nm between 0.01 and 0.05 were prepared for each alkaloid and standard. Their absorption and fluorescence spectra were measured using excitation at 322 nm and an emission range covering the whole emission spectrum of a particular QBA or standard. Inner-filter effect was corrected using Eq (5) [31].

The fluorescence spectra were integrated and plotted against corresponding absorbance at the excitation wavelength. Resulting slopes were used for quantum yield (QY) calculation according to Eq (6), where *Φ* is fluorescence QY, *slope* is slope of integrated fluorescence vs. absorbance plot and *η* is refractive index of the solvent. Subscripts *QBA* and *ST* denote QBA and standard, respectively.

$$\Phi_{QBA}=\Phi_{ST}\cdot \frac{slope_{QBA}}{slope_{ST}}\cdot \frac{\eta_{QBA}^{2}}{\eta_{ST}^{2}}$$ (6)

This is a version of a well-known Eq (7) [26] recognizing that when integrated fluorescence of a sample is plotted as a function of its absorbance at the excitation wavelength, the slope equals fluorescence-to-absorbance ratio.

$$\Phi_{QBA}=\Phi_{ST}\cdot \frac{F_{QBA}}{F_{ST}}\cdot \frac{A_{ST}}{A_{QBA}}\cdot \frac{\eta_{QBA}^{2}}{\eta_{ST}^{2}}$$ (7)

Standards were cross-correlated to find out if measurements give reliable QYs (0.54 for quinine, 0.27 for anthracene [34]). For each QBA, QY was calculated using both standards and the result was obtained by averaging these two calculated values. Each experiment was repeated 4 times and a relative standard deviation (RSD) of 15% was estimated, based on the error of fit and quality of instrument correction factors supplied with the spectrofluorometer.

Fluorescence rate constants were calculated from measured lifetimes and QYs for the alkanolamine form of QBA according to Eqs (8)–(11) [35]:

$$\tau_{S}=\frac{1}{k_{r}^{S}+k_{nr}^{S}}$$ (8)

where *τ*_*S*_ is fluorescence lifetime from singlet excited state, *k*_*r*_^*S*^ is rate constant for radiative deactivation S_1_→S_0_ with emission of fluorescence, *k*_*nr*_^*S*^ is overall nonradiative rate constant (internal conversion plus intersystem crossing) and *Φ*_*F*_ is fluorescence QY.

$$\Phi_{F}=\frac{k_{r}^{S}}{k_{r}^{S}+k_{nr}^{S}}$$ (9)

$$k_{r}^{S}=\frac{\Phi_{F}}{\tau_{S}}$$ (10)

$$k_{nr}^{S}=\frac{1-\Phi_{F}}{\tau_{S}}$$ (11)

### Effect of solvent polarity

Small amounts of stock solutions of QBAs were transferred into Eppendorf tubes or glass volumetric flasks and were evaporated. The solvent (benzene, diethyl ether, methanol, ethanol, octanol, 0.01M borate buffer, pH 9.45) was added to prepare 3 × 10^-6^M solutions. Stokes shifts were determined from absorption (excitation in the case of benzene and diethyl ether) and fluorescence spectra plotted in wavenumber scale using Eq (1). Solvent polarity effect on QBA fluorescence was assessed by Lippert–Mataga plot of Stokes shift against orientation polarizability [35]. Orientation polarizability is defined as (*ε*_*r*_-1)/(2*ε*_*r*_+1)-(*η* ^2^–1)/(2 *η* ^2^+1). Published values of *ε*_*r*_ (relative permittivity) and *η* (refractive index) were used [36].

## Results

### Acid-base properties

**Fig 2:**
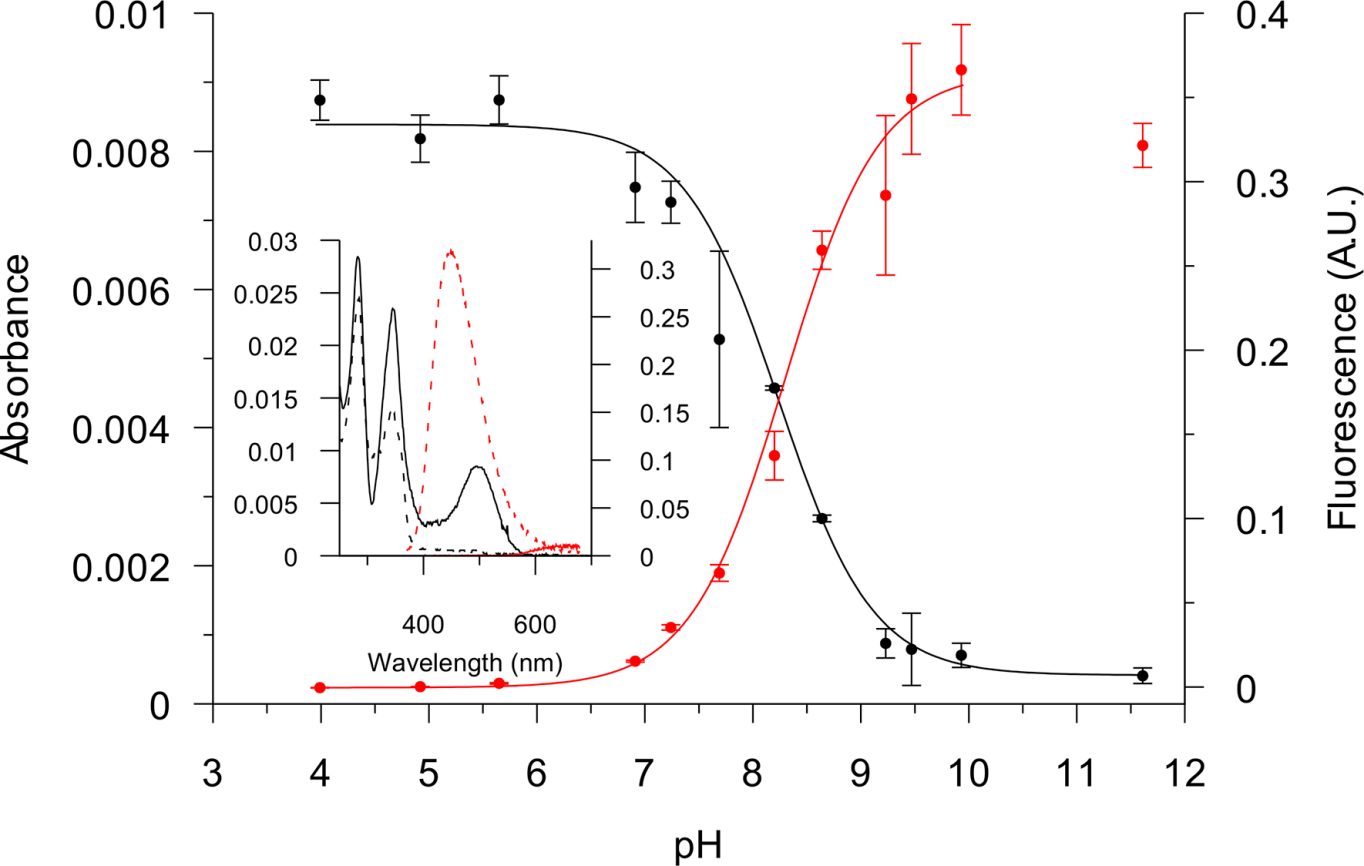
Acid-base properties of 3 μM macarpine. Dependence of absorbance at 495 nm (black) and fluorescence at 450 nm (red) on pH, n = 3. Mean values ± SD and fits to Eq (4) are shown. Inset: Absorption (black) and emission (red) spectra of iminium (—) and alkanolamine (…) form. Ordinates are the same as for bigger figure.

Fig 2 shows absorption and emission spectra of iminium and alkanolamine forms of macarpine and their change with changing pH. It could be seen that the iminium form is generally more absorbing, especially around 500 nm (20000 cm^-1^), but its emission is quenched. The alkanolamine form does not absorb above ca. 400 nm (25000 cm^-1^), but intensely fluoresces at 450 nm (ca. 22220 cm^-1^). In a highly alkaline solution fluorescence starts to decrease and was therefore omitted from fitting. Table 1 shows pK_ROH_ values found by spectrophotometry and spectrofluorometry. Both methods give similar results. It is possible to align the QBAs by increasing pK_ROH_ using published values [30] as follows: chelirubine < sanguirubine < sanguinarine < macarpine < chelilutine < sanguilutine < chelerythrine.

### Fluorescence lifetime

Fluorescence lifetime of QBAs at pH 9.45 (mainly alkanolamine form present) is between 3 and 5 ns (Table 1). Previously reported lifetime for sanguinarine (3.3 ns) [22] is in good agreement with the found value of 3.2 ns. As can be seen from Table 1, addition of ctDNA does not change fluorescence lifetimes of the alkanolamine form (except macarpine).

Fluorescence of iminium form of QBA is quenched except for sanguinarine and chelerythrine, but only the sanguinarine lifetime could be reliably measured. The measured value of 2.4 ns is the same as that reported previously [22,37]. The addition of DNA causes a decrease of sanguinarine steady-state fluorescence. The lifetime of DNA-bound sanguinarine increases to 10.1 ns with another component of 2.4 ns attributable to its free form in solution (S1 Fig). Again, lifetime of sanguinarine-DNA complex is the same as reported earlier [37]. Steady-state fluorescence of other QBAs increases in the presence of DNA. Their fluorescence decays are monoexponential with lifetimes of alkaloid-DNA complexes around 3 ns. The exceptions are chelerythrine and macarpine. Chelerythrine, whose free form is fluorescent, shows double exponential decay similar to sanguinarine. Therefore, it can be argued that a longer lifetime belongs to a complex with DNA and shorter lifetime belongs to free form in solution. In the case of macarpine a double exponential is necessary to fit the decay, but obtained lifetimes are harder to interpret because lifetime of the free form is not measurable. Using the same assumption as for chelerythrine we could assign a shorter lifetime to free macarpine and a longer lifetime to macarpine-DNA complex.

### Fluorescence quantum yields

To achieve minimum spectral overlap of iminium and alkanolamine forms of QBA, pH 3.95 and 9.45 were used in this study. Only QYs of sanguinarine and chelerythrine iminium form could be determined reliably due to the quenched fluorescence of other alkaloids. As can be seen from the values of pK_ROH_ in Table 1, a higher pH would be better to measure the pure alkanolamine form of chelerythrine and sanguilutine. This was not possible due to a decrease of fluorescence above a pH of around 9.5–10 (Fig 2). Table 1 lists QYs of all alkanolamine forms of QBA and of sanguinarine and chelerythrine iminium forms. Iminium forms of other QBAs have too low a fluorescence for QY determination. The iminium form of sanguinarine has an order of magnitude lower QY than the alkanolamine form. For chelerythrine an even larger difference of two orders of magnitude is observed.

### De-excitation rate constants

Using measured fluorescence lifetimes and QYs, radiative and non-radiative rate constants of S_1_ **→**S_0_ transition of the alkanolamine form of QBAs were calculated using Eqs (10) and (11). All radiative constants are of the order 10^7^ s^-1^; non-radiative constants are of the order 10^8^ s^-1^ implying that radiative de-excitation is unfavourable. QBAs could be ordered according to k_r_^S^ from lowest to highest rate constant as macarpine < chelerythrine < sanguilutine < sanguirubine < sanguinarine < chelirubine.

### Effect of solvent polarity

**Fig 3:**
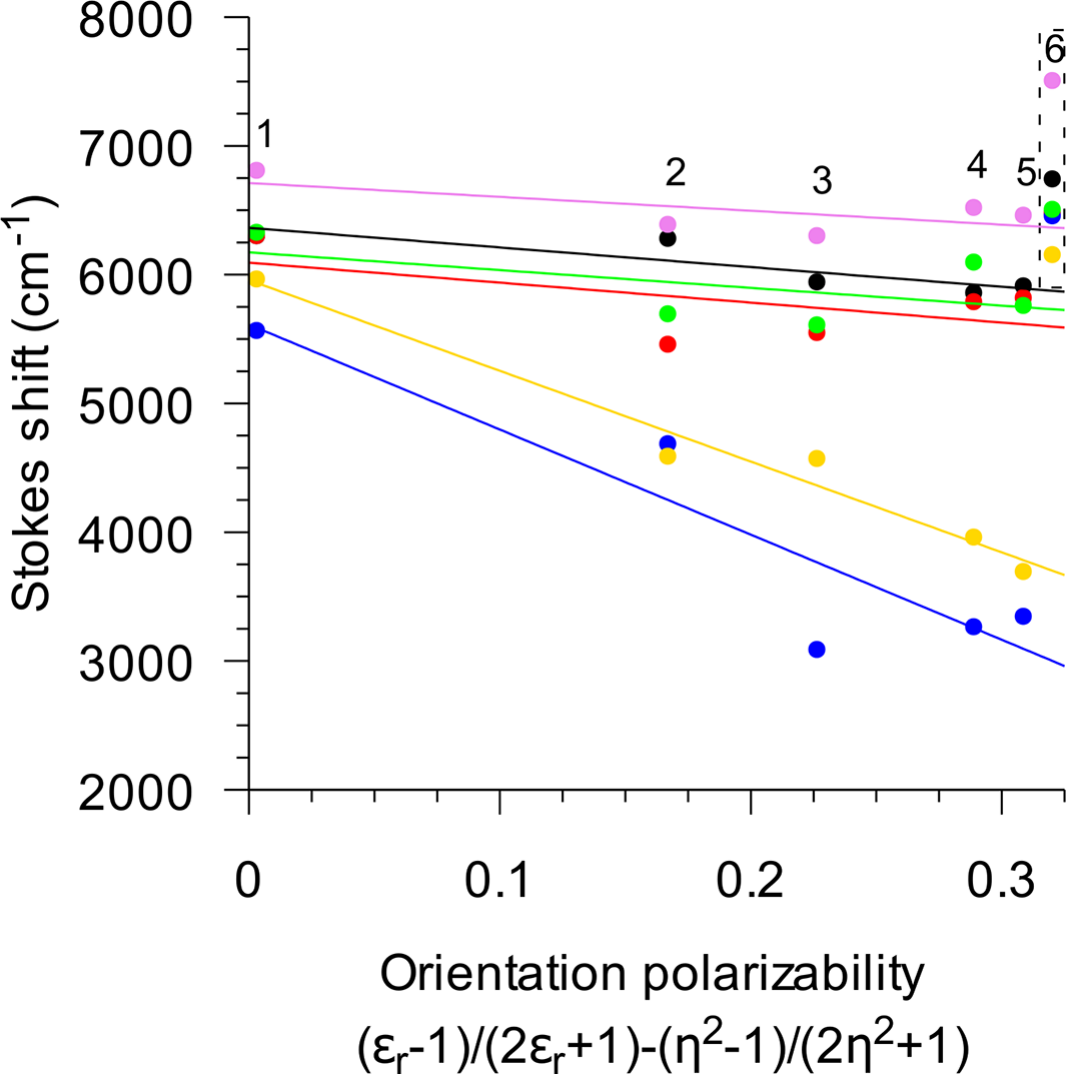
Lippert–Mataga plot of QBAs. Black–macarpine, red–sanguilutine, blue–chelirubine, green–sanguinarine, gold–sanguirubine, violet–chelerythrine; 1 –benzene, 2 –diethyl ether, 3 –octanol, 4 –ethanol, 5 –methanol, 6–0.01M borate buffer, pH 9.45. Samples in borate buffer (dashed box) excluded from fitting.

Stokes shifts in various solvents were analysed by Lippert–Mataga plot. In all studied solvents (benzene, diethyl ether, octanol, methanol, ethanol, 0.1M borate buffer, pH 9.45), spectra of QBAs resemble that of the alkanolamine form with a peak around 430–450 nm (ca. 22000–23000 cm^-1^, S3 Fig). There is a decrease in the Stokes shift between benzene and diethyl ether for all studied QBAs (Fig 3). An interesting effect was observed for spectra in hydrogen-bonding solvents. While methanol, ethanol and octanol decreased Stokes shift in comparison with benzene, borate buffer, pH 9.45 caused an increase.

### Binding to DNA

**Fig 4:**
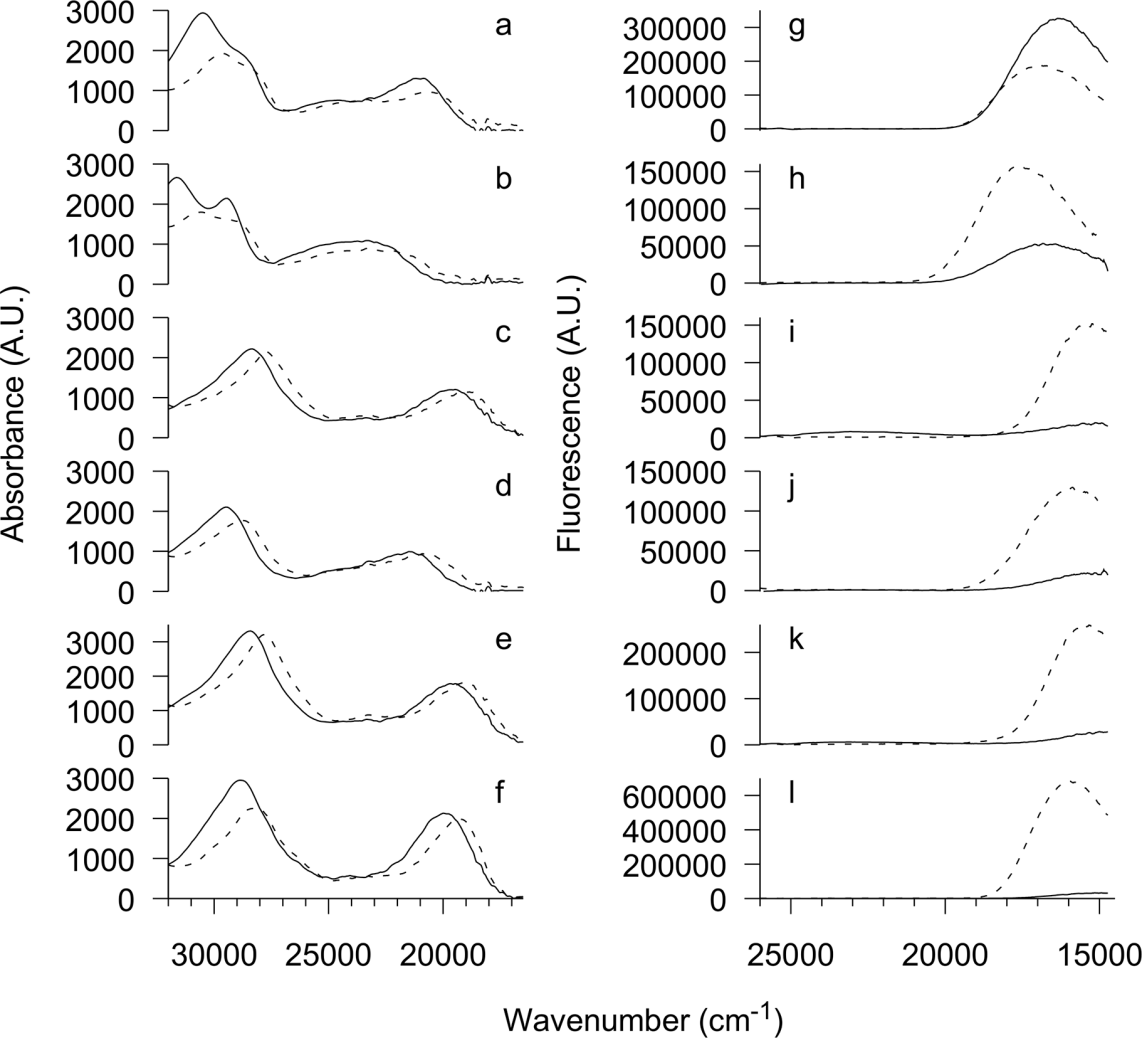
Absorbance and fluorescence spectra of QBAs in absence and presence of ctDNA. QBAs (3 μM in 20mM acetate buffer, 200 mM NaCl, 2mM EDTA, pH 5) in absence (—) and presence (…) of ctDNA (DNA base pair-to-drug ratio 15.9:1). a-f–absorption spectra, g-l–emission spectra; a, g–sanguinarine, b, h–chelerythrine, c, i–chelirubine, d, j–sanguilutine, e, k–sanguirubine, f, l–macarpine. Note that intensities are in arbitrary units due to conversion to wavenumber scale using Eq (1).

Absorption spectra of QBA at pH 5 (Fig 4) show isosbestic points after addition of ctDNA confirming interaction of all studied alkaloids with double-stranded DNA. All QBAs except sanguinarine show increased fluorescence at ca. 16500 cm^-1^ (ca. 600 nm) in the presence of ctDNA. Additional fluorescence peaks of chelirubine and sanguirubine at ca. 23000 cm^-1^ (ca. 435 nm) belong to the alkanolamine form (Fig 4). Another experiment at pH 3.95 (S2 Fig) shows that QBAs in iminium form bind DNA. Absorbance spectra of alkanolamine forms of QBA at pH 9.45 slightly shift to the spectra of iminium-DNA complex in samples with DNA (S2 Fig). The largest shift was observed for macarpine.

**Fig 5:**
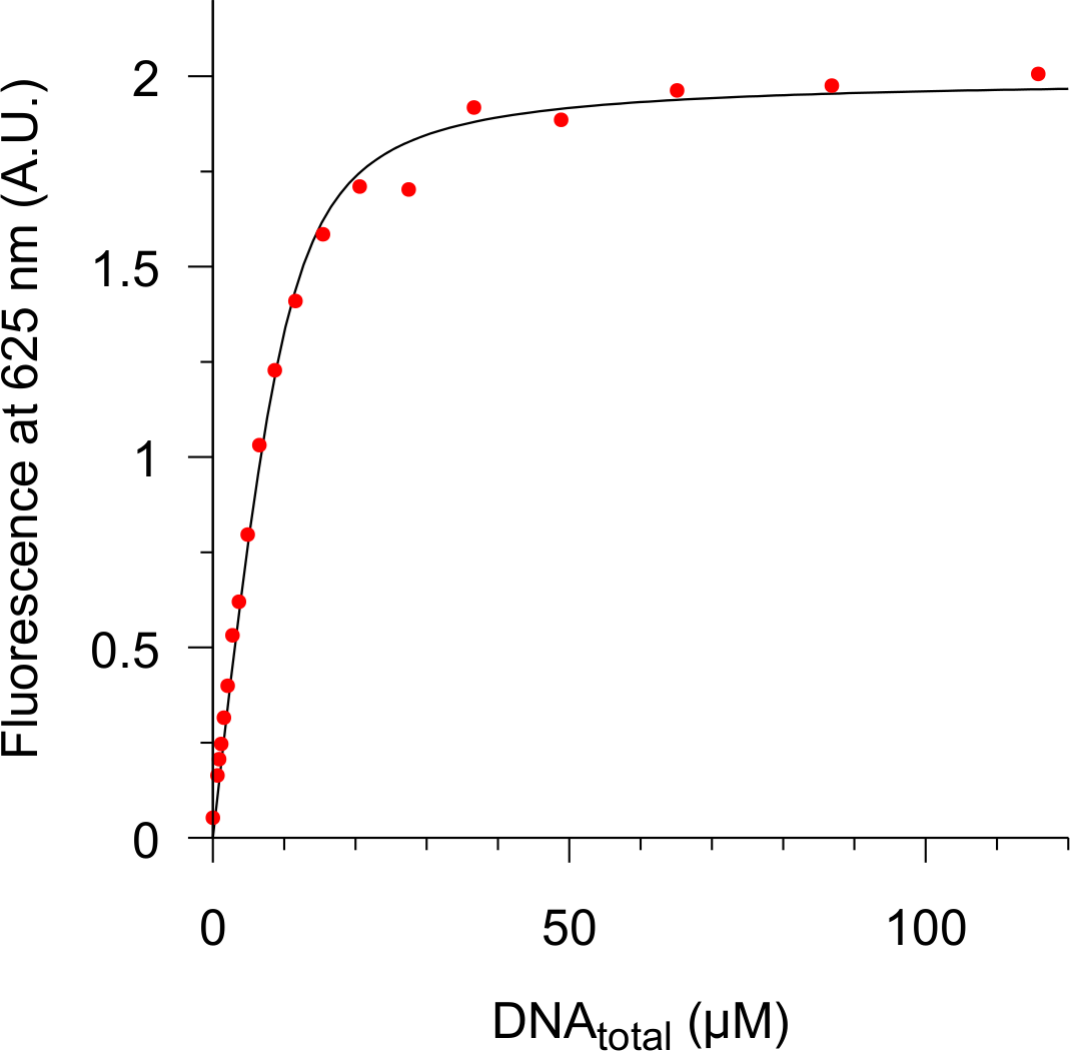
Representative fit of macarpine-DNA interaction. Macarpine (10 μM) binding to salmon testes DNA (0–116 μM bp) in 0.05 M citrate buffer, pH 6.15, [Na^+^] = 0.122 M was measured as a fluorescence change at 625 nm. Data were fitted to 1:1 binding model using DynaFit software.

On the basis of previous experiments, macarpine was selected for further study of DNA interaction. Association constant in a citrate buffer, pH 6.15 with physiologically relevant Na^+^ concentration (122 mM) was measured (Fig 5). Buffer pH was selected to prefer the iminium form of macarpine and stable B-form of DNA. Data were fitted to a 1:1 association constant of 7 × 10^5^ M^-1^ (SD 2 × 10^5^ M^-1^).

According to the energy of iminium ground state (Fig 4) it is possible to order QBAs from lowest to highest: chelirubine ~ sanguirubine < sanguinarine ~ sanguilutine < chelerythrine (both for free and DNA-bound forms). Similarly, energy of the lowest singlet excited state could be ordered as chelirubine ~ sanguirubine ~ sanguilutine < sanguinarine < chelerythrine for free form and chelirubine ~ sanguirubine < sanguilutine < sanguinarine < chelerythrine for the DNA-bound form. It must be added that fluorescence of free chelirubine, sanguirubine, and sanguilutine is highly quenched and not well detected in this experiment so the maximum is not well defined.

## Discussion

### Fluorescence of free and DNA-bound QBAs

Previous studies of sanguinarine and chelerythrine showed that the fluorescence of QBAs comes from two distinct species–neutral alkanolamine and positively charged iminium [20,21]. In aqueous solution typical values of pK_ROH_ for QBAs are between 7.7 (chelirubine) and 9 (chelerythrine) [30]. Our measurement of acid-base equilibrium constant of macarpine and previous experiments of other authors [38] show that spectrophotometry and spectrofluorometry give the same pK_ROH_ within experimental error. This suggests that no photochemical processes take place in an excited state during emission. It could be noted that QBAs with two methoxy substituents at C_7_ and C_8_ have higher pK_ROH_ than QBAs with the methylenedioxy group. Macarpine differs from chelirubine by methoxy substitution at C_12_. This substituent causes an increase of pK_ROH_ of about 0.5.

For iminium form, energies of lowest-energy absorption peak and fluorescence peak are lower for alkaloids with methoxy substitution at C_10_ (chelirubine, sanguirubine, sanguilutine). Furthermore, alkaloids with methylenedioxy substituent at C_7_ and C_8_ have lower energies of the lowest-energy absorption peak and fluorescence peak than alkaloids that differ from them only in methoxy substituents at these positions (chelirubine, sanguirubine vs. sanguilutine, sanguinarine vs. chelerythrine). The same trends can be seen for DNA-bound form of alkaloids. These findings are in accordance with the general effects of electron-donating methoxy and methylenedioxy substituents described for a wide group of isoquinoline alkaloids to which QBAs belong [38].

Changes in lifetimes, absorption, and fluorescence spectra of iminium form of QBAs with the addition of a small excess of DNA show that this form of QBA is able to bind DNA. Lifetimes of alkanolamine form of QBAs were not changed with the addition of DNA and absorption spectra were slightly shifted to the iminium form for all QBAs except macarpine. This suggests that there is no interaction between alkanolamine form of QBAs and DNA. In the case of macarpine, the absorption spectrum was almost totally converted to the spectrum of iminium-DNA complex with the addition of ctDNA. Lifetime is also changed with the addition of DNA under these conditions and similar lifetimes are obtained by setting monochromator to detect iminium form (610 nm). That means that only macarpine is able to convert itself to iminium form and bind to DNA under unfavourable conditions (pH 9.45) at low DNA-to-drug ratio (1.6 in this experiment, expressed as base-pairs). The conversion of alkanolamine to iminium form of sanguinarine in the presence of a large excess of DNA was previously observed by Sen *et al*. [10]. The extent of the shift between iminium and alkanolamine in presence of DNA should depend on both pK_ROH_ and DNA binding constant of the particular QBA. The association constant of macarpine found in this study is 7 × 10^5^ M^-1^. This could be compared with the intrinsic binding constants published previously recognizing that *K* = *K*_*i*_/*n*, where *K* is the association constant used in this study, *K*_*i*_ the intrinsic binding constant and *n* is the size of the binding site [39]. Sen *et al*. found *K*_*i*_ 9.35 × 10^5^ M^-1^ and *n* 3.7 by spectrophotometry and *K*_*i*_ 9.5 × 10^5^ M^-1^ and *n* 3 by spectrofluorometry [10] for interaction with ctDNA. That is about a half of the association constant of macarpine. Methoxy substituent at C_12_ of the macarpine molecule is probably responsible for a much tighter binding to DNA.A previous binding experiment did not result in the highest binding constant among QBAs, but the shape of the binding isotherm did not show saturation [24]. No nonspecific binding was observed in the present study. The difference could be explained by a different concentration of Na^+^–physiologically relevant concentration of 122 mM Na^+^ compared to 50 mM in the previous study [24]. A higher concentration of Na^+^ could supress any nonspecific interaction of a charged iminium form of QBA with a negatively charged DNA backbone. The sanguinarine iminium form binds to double-stranded DNA by intercalation, as was confirmed by lengthening of the DNA contour length [10,40]. Based on the structural similarity and analogous spectral behaviour of other QBAs in the presence of double-stranded DNA intercalation is expected to be the main mode of binding. Due to the charge of the iminium form electrostatic interaction is another mode of binding that is probably present, but its influence is rather limited. Calorimetric studies of sanguinarine showed that the contribution of electrostatic forces to binding free energy is below 15% [41].

### Quantum yields and de-excitation rate constants

QY estimations of QBAs at pH 5.3 has been previously published [24]. At this pH minute amounts of more fluorescent alkanolamine form complicate determination of the iminium form QY. Therefore, our data collected at pH 3.95 and 9.45 resulted in different QYs. The iminium form of QBAs is quenched, except for sanguinarine and chelerythrine, so it was not possible to measure QYs of these QBAs. Almost the same value for sanguinarine iminium QY (0.0036) has been previously published [42]). Alkanolamine QY of sanguinarine published in that article was lower than that found in this work (0.134 vs. 0.210). This could be caused by quenching of fluorescence at an alkaline pH (Fig 2) so apparent QY would be pH-dependent. Only part of the quenching could be explained by dynamic quenching by hydroxide anions, because the lifetime at pH 11.43 is only 0.1 ns lower than that below pH 9.45 (S4 Fig). Photochemical conversion of the sanguinarine alkanolamine form to non-fluorescent oxysanguinarine has been previously reported [43] and could be the cause of observed reduction of fluorescence.

Differences of alkanolamine QYs could be found between structurally related sanguinarine, chelirubine, and macarpine. QY of sanguinarine is similar to other QBAs. Chelirubine, which differs from sanguinarine only in methoxy substituent at C_10_, has the highest QY of all QBAs. Contrastingly, macarpine, which differs from chelirubine only by additional methoxy substituent at C_12_, has the lowest QY that is about 3 times lower than that of chelirubine. The position of this substituent has to be important for preference of non-radiative over radiative decay of the alkanolamine form.

Rate constants of de-excitation processes were calculated from lifetimes and QYs of alkanolamine forms of QBAs on the assumption that fluorescence from S_1_ is the only radiative process. All radiative rate constants of QBAs were of the order 10^7^ s^-1^ while non-radiative constants were of the order 10^8^ s^-1^ implying that radiative de-excitation is unfavourable. Apart from macarpine with its substituent on C_12_, other alkaloids show a trend. Alkaloids with methylenedioxy substituent at C_5_ and C_6_ have higher rate constants than alkaloids with two methoxy substituents.

QYs of alkaloid-DNA complexes were not determined, because it would need a complete conversion of free alkaloid to the bound form. However, it is possible to assess changes of QYs from changes of fluorescence intensity upon DNA binding. While fluorescence of sanguinarine decreases, other QBAs show an increase upon DNA binding with macarpine being the most fluorescent QBA in the complex with DNA. This indicates that methoxy substituent at C_12_ improves not only binding, as was shown above, but also improves fluorescent properties of the DNA-bound complex. Together with the lowest QY of alkanolamine form these properties make macarpine a potential DNA probe. The use of QBAs for DNA staining and flow cytometry has already been published with macarpine being the only QBA to allow quantitative cell-cycle analysis [19]. Our results could explain this behaviour as a result of the good fluorescence signal of macarpine-DNA complex and a high affinity of macarpine to DNA over a wide range of pH. Further research into the thermodynamics of macarpine-DNA binding could reveal why C_12_ substitution positively influences its binding properties.

### Solvent effects

Influence of solvent polarity on fluorescence spectra is well-known and could be used to probe microenvironment around a fluorescent molecule. A Lippert–Mataga plot could be used to assess changes of Stokes shift as a result of different orientation polarizability of solvents. Without any interaction between solvent and solute this plot usually results in a straight line.

Sanguinarine spectral shifts have been previously analysed by this method [23]. Even though a different set of solvents was used a similar result was obtained *i*.*e*. only a small effect of non-aqueous solvents on Stokes shift. Alcohols are known to react with the iminium bond of QBA in a similar way to hydroxide [44]. Therefore, a large difference in Stokes shift between water and the studied alcohols could be attributed to the different effect of hydroxy, methoxy, ethoxy, and octyloxy substituents on the ground and excited state of QBAs. Hydroxy, methoxy, ethoxy, and octyloxy substituents are all strong electron-donating groups so the observed difference could be caused by a different effect of hydroxyl substituent and hydrophobic aliphatic chains of alcohols. Another possible contribution to this difference might be the different hydrogen-bonding capability of alcohols and water. While it is not possible to correlate different slopes of QBAs in a Lippert–Mataga plot to their structure, sanguirubine and chelirubine, whose slopes deviate from other QBAs, also have very similar absorption and fluorescence spectra. Therefore, it could be concluded that different substituents on C_2_ and C_3_ (sanguirubine–two methoxy groups, chelirubine–one methylenedioxy group) have only a small effect on the positions of peaks and susceptibility to various solvents, but as was shown above there is an effect on QYs and fluorescence lifetimes.

## Conclusion

All studied QBAs have two forms depending on pH–more fluorescent alkanolamine and quenched iminium. The alkanolamine form is also present in non-polar media and similar spectra are found for alcoholic adducts. It was shown that only the iminium form of studied QBAs interact with double-stranded DNA. This interaction is accompanied with an increase of fluorescence for all QBAs except sanguinarine and with prolongation of fluorescence lifetime. Macarpine, the only QBA with substitution on C_12_, was found to be able to bind DNA over a wide range of pH. It has the highest increase of iminium form fluorescence upon binding to double-stranded DNA and the lowest fluorescence of the alkanolamine form. Therefore, further study of its possible use as a fluorescent DNA probe would be beneficial.

## Supporting Information

S1 Fig Fluorescence lifetimes of both forms of sanguinarine in absence and presence of ctDNA. DNA base pair:drug ratio 1.6:1, IRF–instrument response function.
(TIFF)

S2 Fig Absorption spectra of QBAs in acidic and basic environment in presence and absence of ctDNA. SG–sanguinarine, CHE–chelerythrine, CHR–chelirubine, MA–macarpine, SL–sanguilutine, SR–sanguirubine.
(TIFF)

S3 Fig Representative emission spectra of macarpine in various solvents. Black–benzene, red–diethyl ether, blue–octanol, green–ethanol, gold–methanol, violet – 0.01M borate buffer, pH 9.45. Note that spectra in methanol, ethanol, and octanol are overlapped.
(TIFF)

S4 Fig Influence of high pH on fluorescence lifetime of sanguinarine. (TIFF)

S5 Fig DynaFit fitting script. Script used for fitting of the first binding experiment between macarpine and DNA. Fitting of the other two experiments was done in the same way.
(TIFF)

S6 Fig Second replication of macarpine-DNA interaction experiment. Macarpine (10 μM) binding to salmon testes DNA (0–116 μM bp) in 0.05 M citrate buffer, pH 6.15, [Na^+^] = 0.122 M was measured as a fluorescence change at 625 nm. Data were fitted to 1:1 binding model using DynaFit software.
(TIFF)

S7 Fig Third replication of macarpine-DNA interaction experiment. Macarpine (10 μM) binding to salmon testes DNA (0–116 μM bp) in 0.05 M citrate buffer, pH 6.15, [Na^+^] = 0.122 M was measured as a fluorescence change at 625 nm. Data were fitted to 1:1 binding model using DynaFit software.
(TIFF)
