# Supplementary figures and images for: Influence of Solvent Polarity and DNA-Binding on Spectral Properties of Quaternary Benzo[c]phenanthridine Alkaloids
Source: PLoS One. 2015 Jun 19;10(6):e0129925. doi: 10.1371/journal.pone.0129925 (PMC4474729; doi:10.1371/journal.pone.0129925)

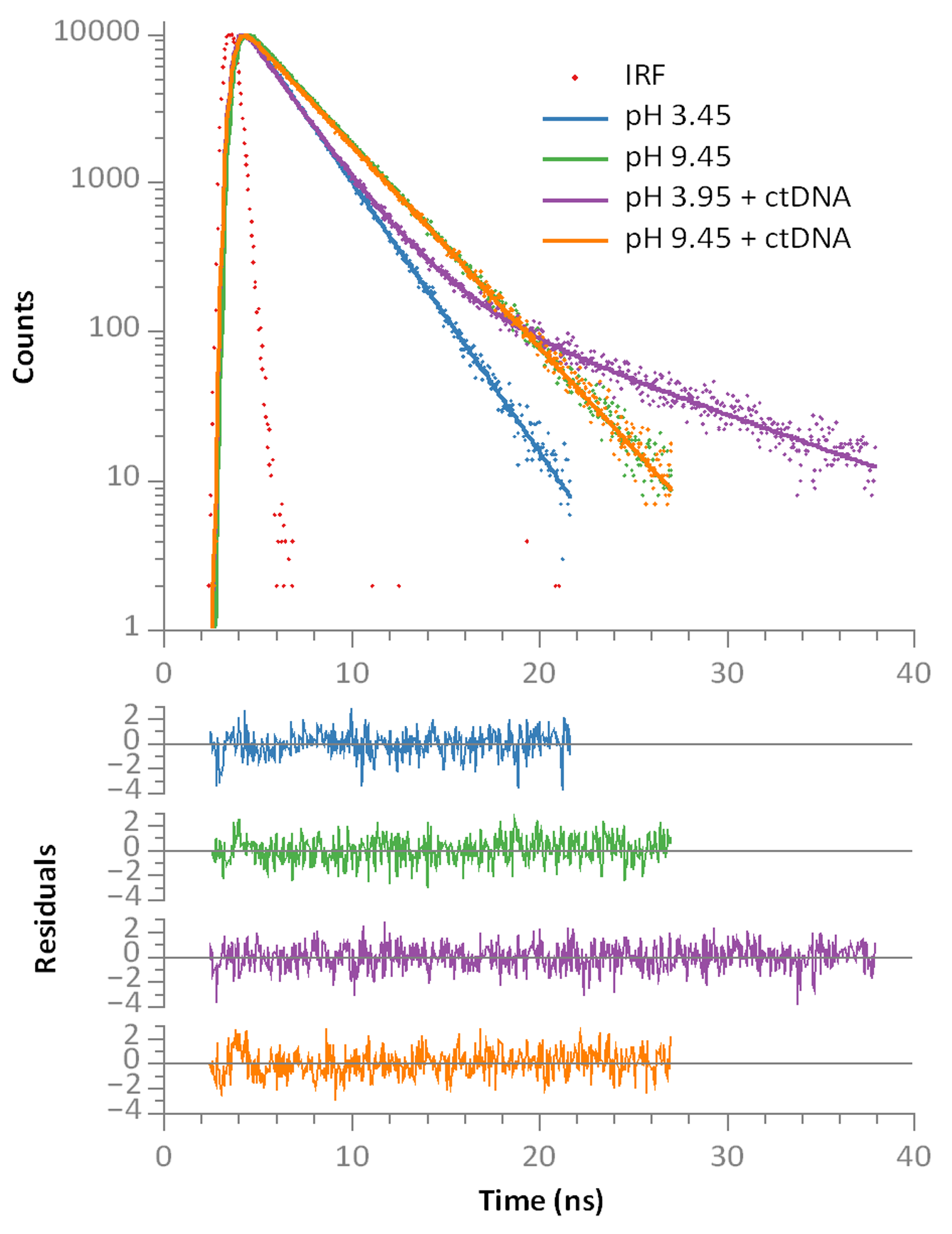

Supplement: S1 Fig — DNA base pair:drug ratio 1.6:1, IRF–instrument response function. (TIFF) [file pone.0129925.s001.tiff]

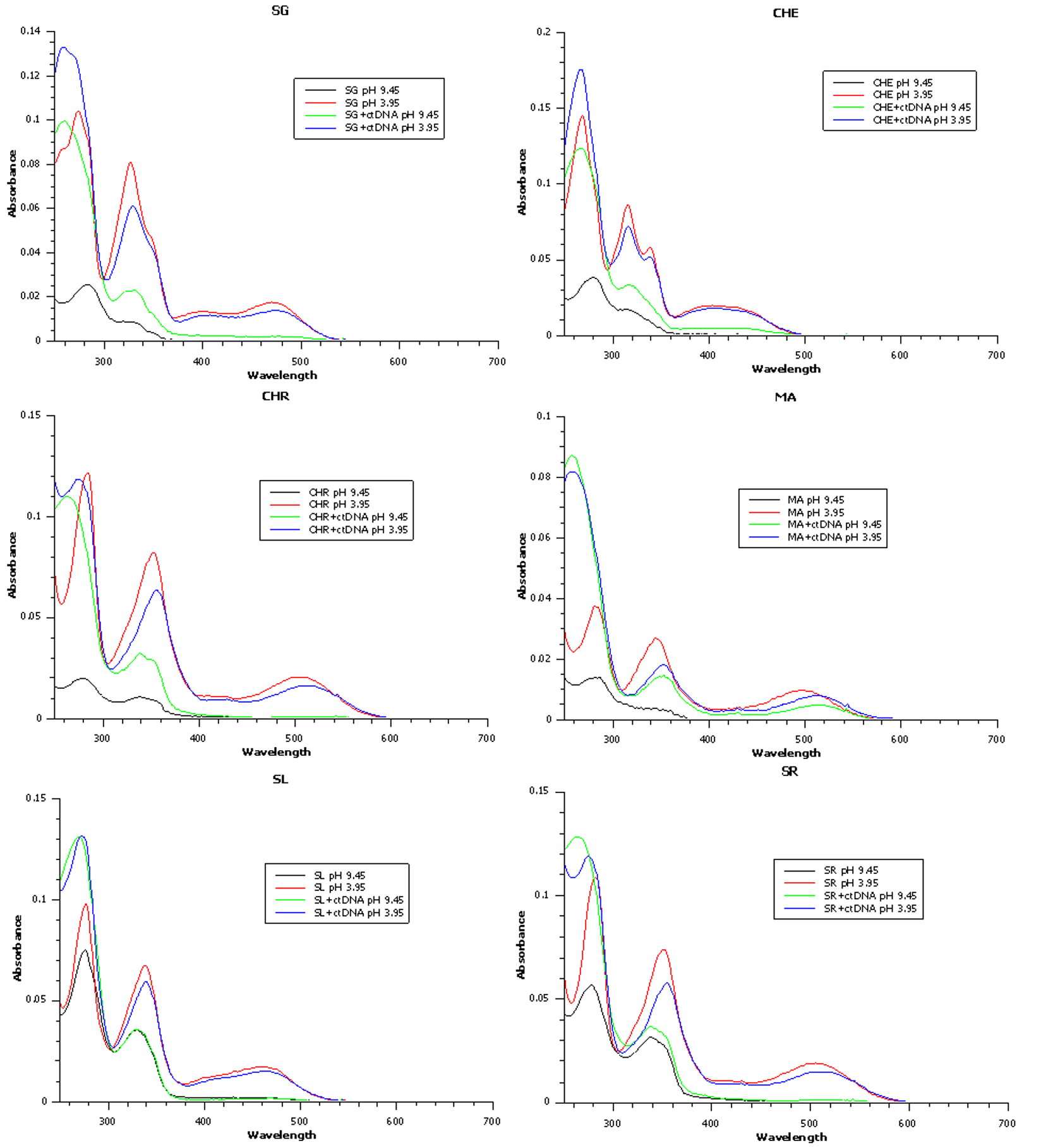

Supplement: S2 Fig — SG–sanguinarine, CHE–chelerythrine, CHR–chelirubine, MA–macarpine, SL–sanguilutine, SR–sanguirubine. (TIFF) [file pone.0129925.s002.tiff]

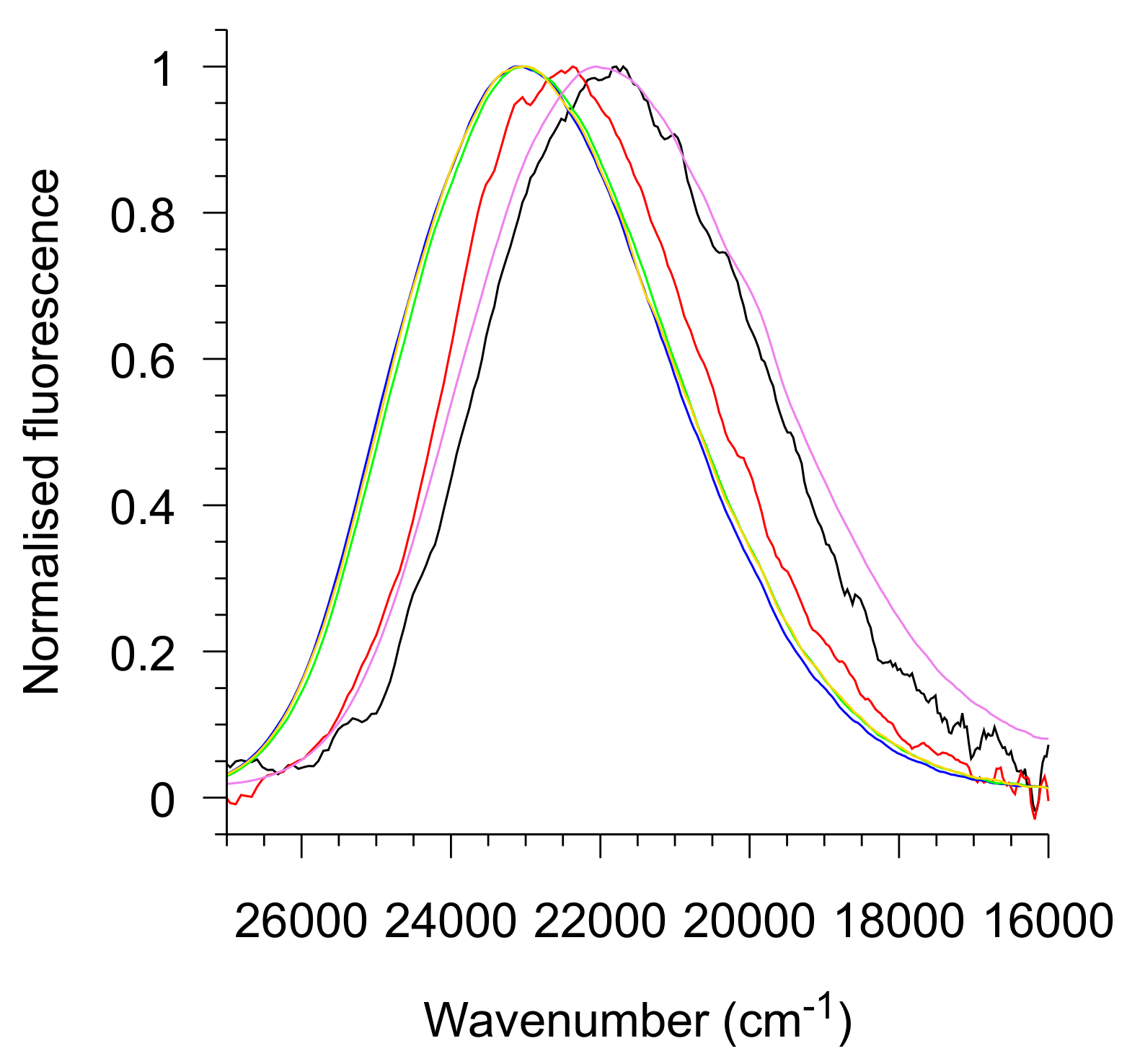

Supplement: S3 Fig — Black–benzene, red–diethyl ether, blue–octanol, green–ethanol, gold–methanol, violet – 0.01M borate buffer, pH 9.45. Note that spectra in methanol, ethanol, and octanol are overlapped. (TIFF) [file pone.0129925.s003.tiff]

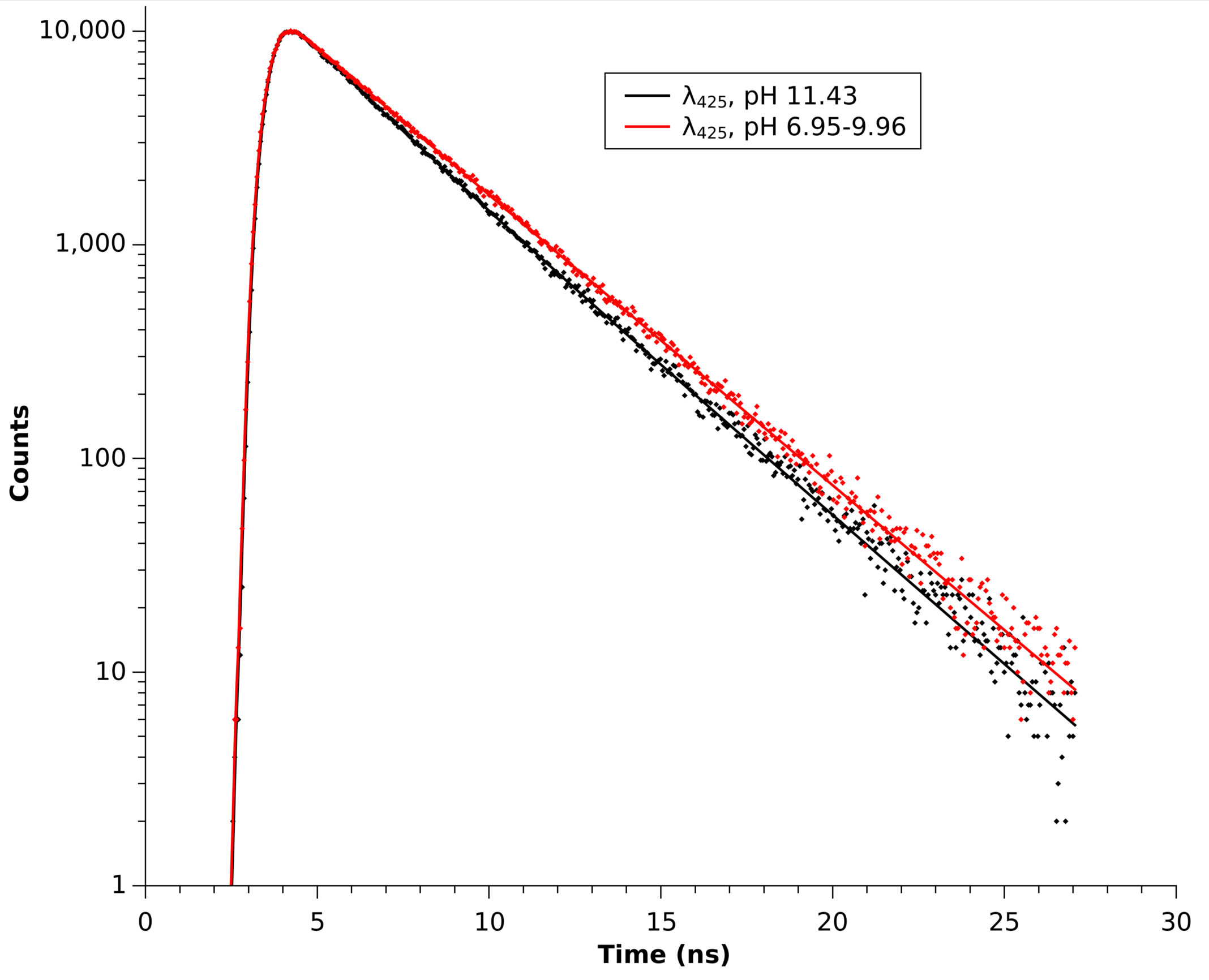

Supplement: S4 Fig — (TIFF) [file pone.0129925.s004.tiff]

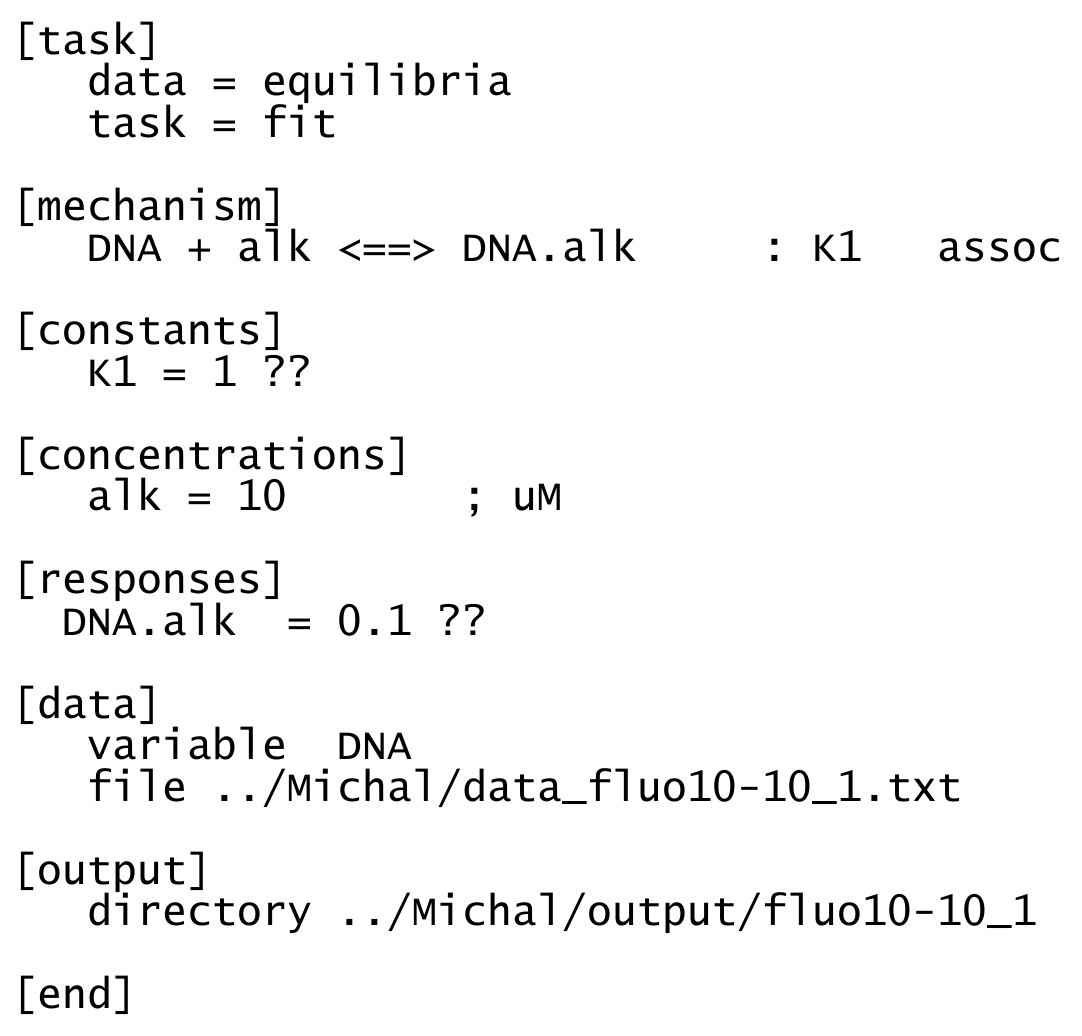

Supplement: S5 Fig — Script used for fitting of the first binding experiment between macarpine and DNA. Fitting of the other two experiments was done in the same way. (TIFF) [file pone.0129925.s005.tiff]

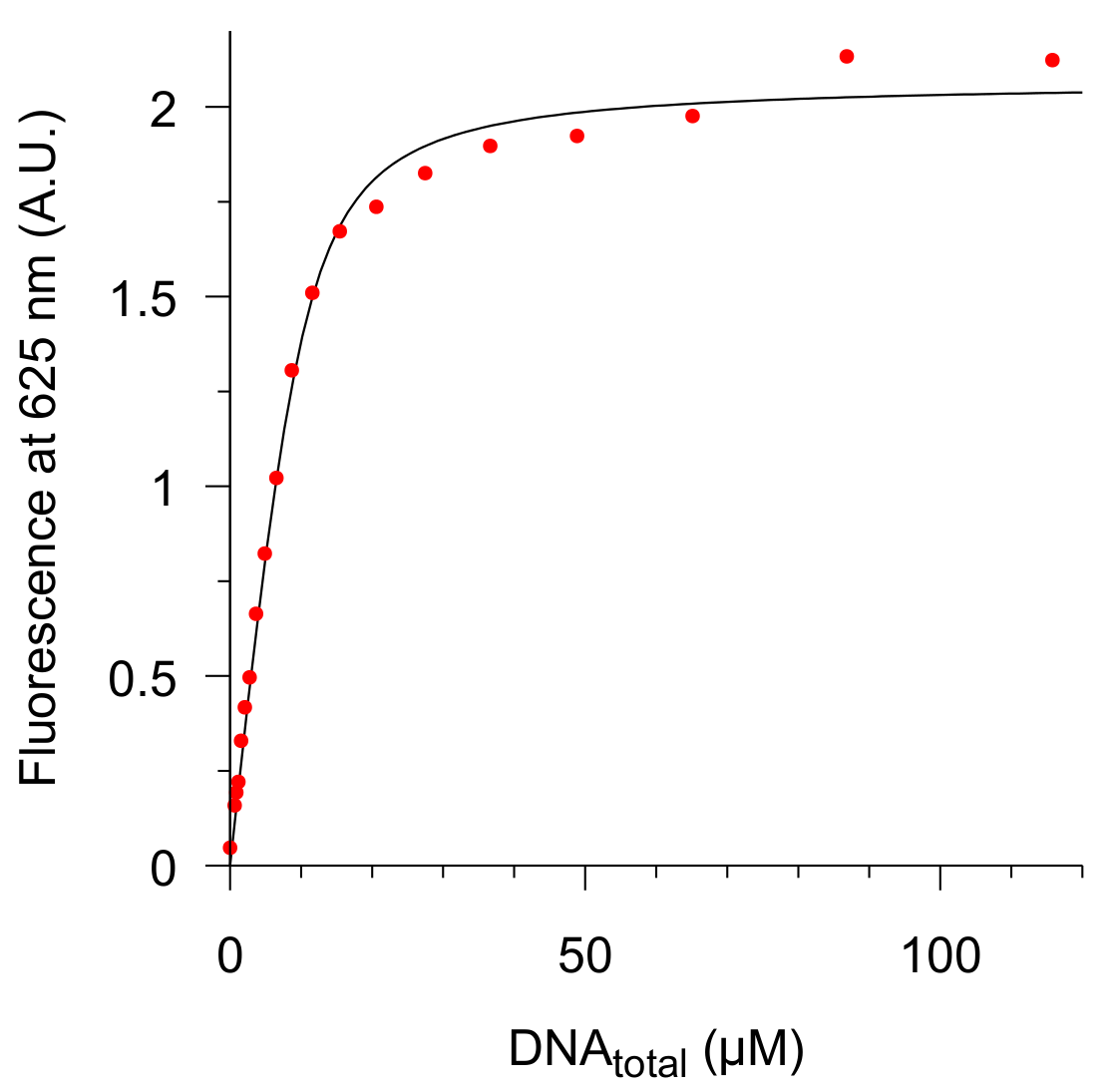

Supplement: S6 Fig — Macarpine (10 μM) binding to salmon testes DNA (0–116 μM bp) in 0.05 M citrate buffer, pH 6.15, [Na+] = 0.122 M was measured as a fluorescence change at 625 nm. Data were fitted to 1:1 binding model using DynaFit software. (TIFF) [file pone.0129925.s006.tiff]

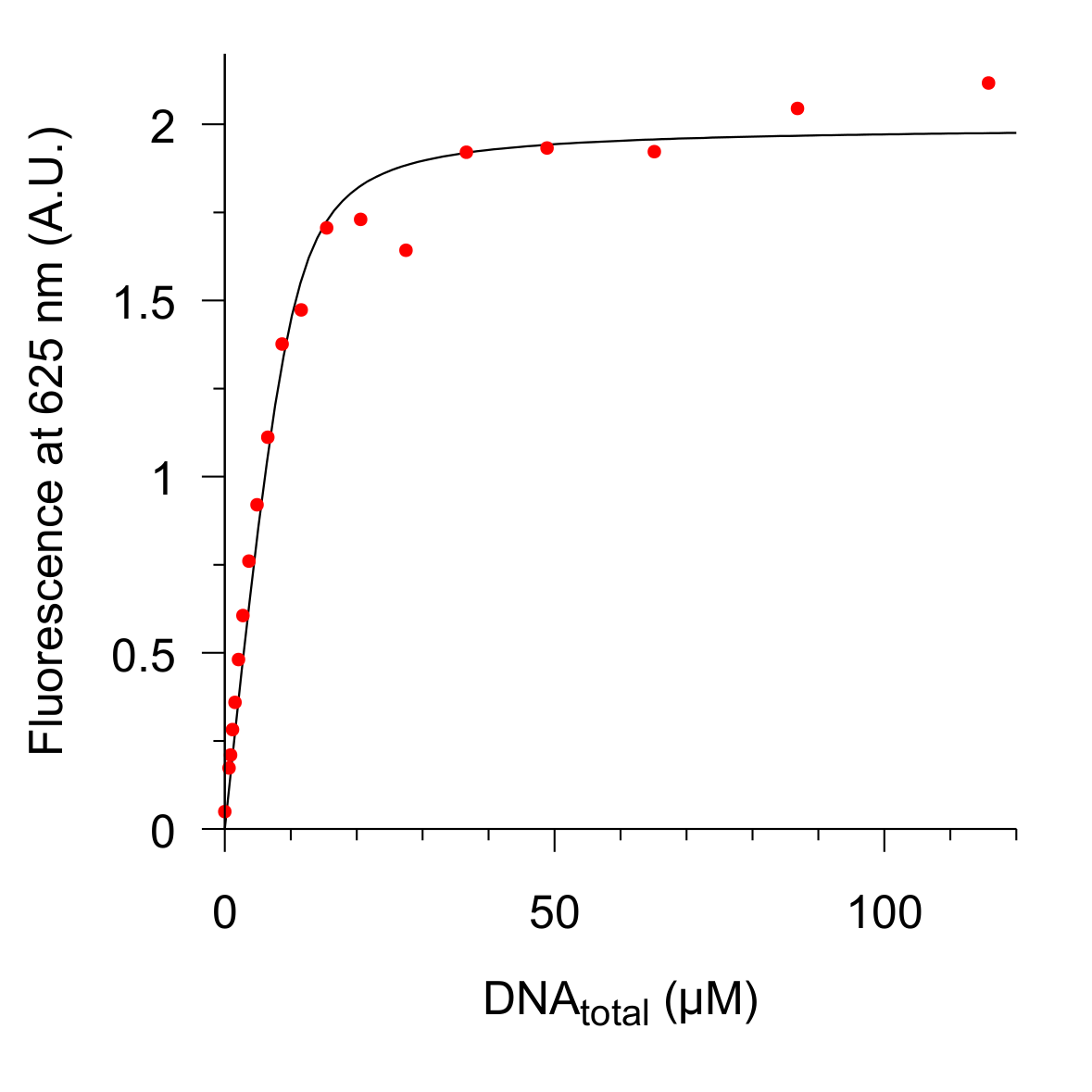

Supplement: S7 Fig — Macarpine (10 μM) binding to salmon testes DNA (0–116 μM bp) in 0.05 M citrate buffer, pH 6.15, [Na+] = 0.122 M was measured as a fluorescence change at 625 nm. Data were fitted to 1:1 binding model using DynaFit software. (TIFF) [file pone.0129925.s007.tiff]
